# Extensor tendon ruptures in rheumatoid wrists

**DOI:** 10.1007/s00590-020-02731-1

**Published:** 2020-07-03

**Authors:** C. Biehl, M. Rupp, S. Kern, C. Heiss, T. ElKhassawna, G. Szalay

**Affiliations:** 1 Klinik Und Poliklinik für Unfall-, Hand- und Wiederherstellungschirurgie-Operative Notaufnahme, UKGM Universitätsklinikum Gießen Und Marburg, Rudolf-Buchheim-Str. 7, 35392 Giessen, Germany; 2 Experimentelle Unfallchirurgie, Justus-Liebig-Universität Giessen, Aulweg 128, ForMED (Forschungsgebäude Medizin), 35392 Gießen, Germany

**Keywords:** Extensor tendon, Tendon ruptures, Rheumatoid arthritis, Wrist function, Tendon reconstruction, Tendon suture

## Abstract

**Background and aims:**

Rheumatoid arthritis is a chronic inflammatory disease. The associated involvement of hands and tendons is over 90% and impairs overall function. In the course of the disease, the joints are often operated on. During this operation, ruptures of the extensor tendons are found by chance without the patients noticing them. The aim of this retrospective study is the prevalence of extensor tendon rupture. Which tendon is destroyed most frequently? How can the functional outcome be measured after reconstruction?

**Materials and methods:**

From 1572 operations on rheumatoid wrists, 61 extensor tendon ruptures were identified in 41 patients. The average time between the first rheumatic symptoms of the hand and surgery was 6.4 years. The average duration of RA was 7.8 years. 26 patients with 27 tendon reconstructions were included in the follow-up with an average postoperative duration of 4.6 years (3 to 14.2 years).

**Results:**

Extensor tendons ruptures typically occurred at mechanically stressed sites. The most frequent rupture was found in the extensor pollicis longus tendon (21 tendons), followed by the small finger extensor tendon (14 tendons). A transfer was performed on 7 tendons. Fifty-five tendon lesions were sutured at other intact tendons. Free grafts were not used. The results in Clayton and QuickDASH scores were significantly different. Functional improvement was consistent with the results of tendon reconstructions in healthy control groups.

**Conclusion:**

In rheumatoid patients, a rupture of an extensor tendon must be expected at 4%. Patients tolerate and compensate this damage for a long time. The function of the hand including the tendon function is the most important factor in assessing the success of the operation. The subjective patient acceptance depends on the progress of the underlying disease, postoperative care (ergotherapy, physiotherapy, orthosis) and the patients' demands.

**Electronic supplementary material:**

The online version of this article (10.1007/s00590-020-02731-1) contains supplementary material, which is available to authorized users.

## Introduction

**Fig. 1:**
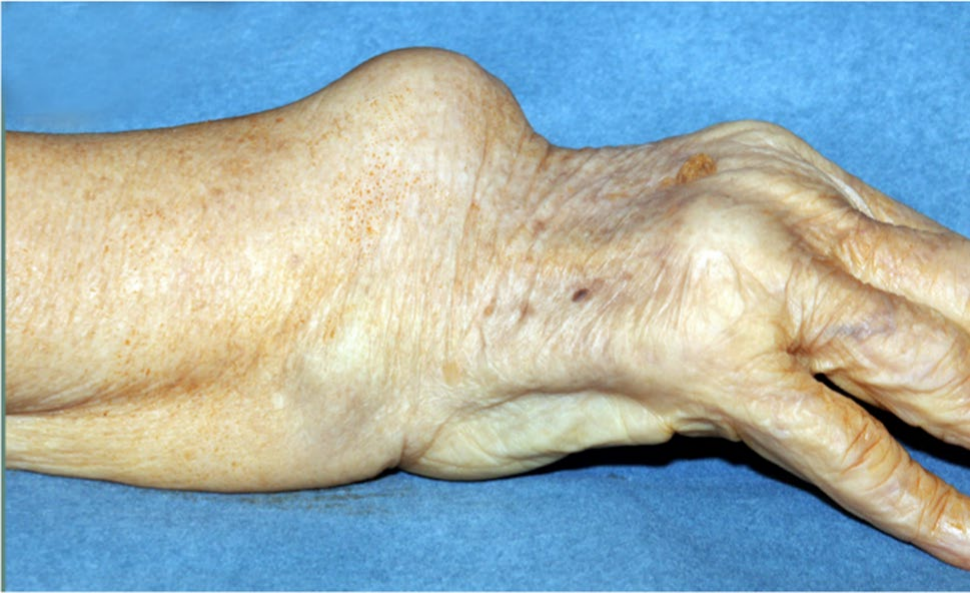
Clinical aspect of the involvement of extensor tendon in rheumatoid wrist with swelling on the dorsal side and lack of extension in the MCP—joint line

Rheumatoid arthritis (RA) can lead to extensor tendon ruptures. They are not always clinically apparent, especially when peritendinous synovia lead to adhesion of the tendon ends. Malpositioning of the fingers and often painful restrictions of movement are long-term effects. These can lead to ankylosis and affect other fingers and the use of the entire hand [16]. At the same time, many rheumatoid patients become accustomed to the slowly developing limitations (Fig. 1). Statements about the frequency of extensor tendon ruptures at the wrist level (zone VII) are rare. Ehrlich et al. [7] already described individual cases in 1959. Surgeons knew the predilection sites for a tendon rupture at the wrist, the Lister tubercle for the extensor pollicis longus tendon. The destroyed ulna head and the triangular fibrocartilage complex leads to the destruction of the tendon of extensor digiti minimi and extensor carpi ulnae (Fig. 1). Williamson et al. [21] found a prevalence of 1.6% of ruptured EDM tendon in rheumatoid wrists. Moore et al. [11], Nakamura et al. [12], and Sakuma et al. [18] report larger case numbers and most publications show significantly fewer case numbers.

Surgical treatment aims to improve the function of the wrist. The overall function of the hand and fingers depends on the good function of the tendons. A specific score for finger tendons or finger function does not exist. The known scores (Clayton, Funktionsfragebogen Hannover—functional questionnaire Hannover (FFbH), Activity of Daily Life (ADL), QuickDASH) record the overall situation of hand and arm, but do not differentiate further. In the cohort, patients achieve only below-average scores. The reason for this was seen in the accompanying tendon pathology.

Therefore, the specific benefit for tendon reconstruction or individual finger function is difficult to distinguish based on the mentioned scores.

This study aimed to determine the prevalence of extensor tendon ruptures in rheumatoid wrists and to make statements about the outcome after reconstruction.

## Patients and methods

This retrospective study includes the treatment of extensor tendon ruptures of the wrist. All patients gave written consent to participate, and ethical approval was not required (Regional Ethics Committee for Rhineland-Palatinate; Mainz, Germany).

Between 1984 and 2015, 1572 rheumatoid wrists were surgically treated. An extensor tendon reconstruction was necessary for both acute symptomatic and hidden extensor tendon ruptures during wrist surgery. Postoperatively, the early exercise of the tendons was performed by an ergotherapist. Also, a dynamic therapy with an inverted "Kleinert" extension splint was performed. This causes relief of the tendon suture and at the same time enables a protected movement and had to walk for at least 6 weeks. Afterward, a free extension of the fingers was made possible.

Patients were questioned about their limits in everyday life directly preoperatively and at time of follow-up (12 weeks after the operation) and clinically examined as well. This was monitored using an adjusted score for everyday functions (FFbH), the QuickDASH score, and a 100-point Clayton score (mobility, stability, ligament tension, and pain) [5, 8–10, 15, 19]. Only the Clayton Score provides a measuring instrument for the physician. The other scores (FFbH, QuickDASH) are based exclusively on the patient's information. During the follow-up, the authors examined the parameters of pain, strength, range of motion, and everyday functions.

However, due to the small group size and the lack of standard distribution, the statistical evaluation of the nonparametric procedures was performed with the Mann–Whitney *U* test and Kolmogorov–Smirnov test. The significance level was set at *p* ≤ 0.05 for all analyses. ANOVA and Bonferroni correction were used as controls.

## Results

In 41 patients, 62 extensor tendon ruptures at wrist level were detected after synovectomy of the tendon, with necessary tendon suture. The incidence of a tendon rupture in our cohort is 3.9% (62/1572).

The average age of patients at surgery was 62.2 (range, 32–92) years, the average duration of wrist pathology was 6.4 (range, 2–5) years. The average duration of RA was 7.8 years.

Of these extensor tendon reconstructions, 26 patients with 27 tendon surgeries were examined at follow-up. Five patients had already died at the follow-up. Nine patients were no longer identifiable or refused further contact with the department. This corresponds to a responder rate of 66% of surgically treated wrists (27/41). The average follow-up was 4.6 (range, 33–14.2) years postoperatively.

**Fig. 2:**
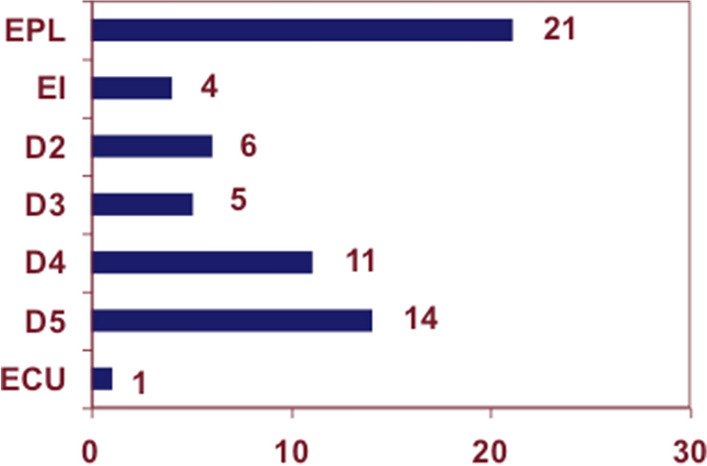
Extensor tendon ruptures, broken down to finger tendon

The extensor tendons were pathologically altered with varying frequency. The question is how high the prevalence of tendon comorbidity of ruptured tendons is. A rupture of the extensor pollicis longus tendon (extensor policis longus: EPL) (21 tendons) was diagnosed most, followed by the small finger extensor tendon (extensor digiti minimi: EDM) (14), the ring finger (11), the index finger (6) and the middle finger (5). The extensor indicis (EI) was affected 4 times, a rupture of the extensor carpi ulnaris tendon (ECU) only once (Fig. 2).

Of 62 extensor tendons, we required 7 primary reconstructions and 55 couplings with other tendons (e.g., EI > EPL). If the 4th and 5th tendons were ruptured, they were sutured with other tendons. However, the tendon of the middle finger was used. No free tendon grafts had to be used. No revisions were necessary. Several tendons had to be reconstructed. The risk of concomitant, additional tendon damage with a tendon rupture was 51%.

The Patient-Reported Outcome Measures (PROMs) on the general everyday function postoperative hardly differed from the preoperative values. The authors asked specific hand and finger function according to the scores of ADL/FFbH and QuickDASH. Similar to in assessing subjective power. However, the patients rated the tendon function significantly better than the total function of the hand.

The Clayton Score increased from 49.6 points preoperatively to 60.1 points postoperatively (max. 100 points; *p* = 0.072), which is still a poor result. The Clayton Score differed significantly from QuickDASH in the postoperative values (*p* = 0.038). In the Functional Questionnaire Score, the patients achieved an average of 20 points postoperatively (FFbH, max. 40 points; *p* = 0.102). In terms of content, QuickDASH and ADL / FFbH ask for parameters regarding the usability of the hand. The questions in both evaluations are 70% identical. The results of the Functional Questionnaire Score (ADL / FFbH) were inversely proportional to the results of the Clayton Scores (no significance, *p* = 0.72) and QuickDASH (*p* ≤ 0.01).

In nine patients, the scores worsened in the evaluation, in one patient they remained unchanged. Seventeen patients showed better results at follow-up (63%).

**Fig. 3:**
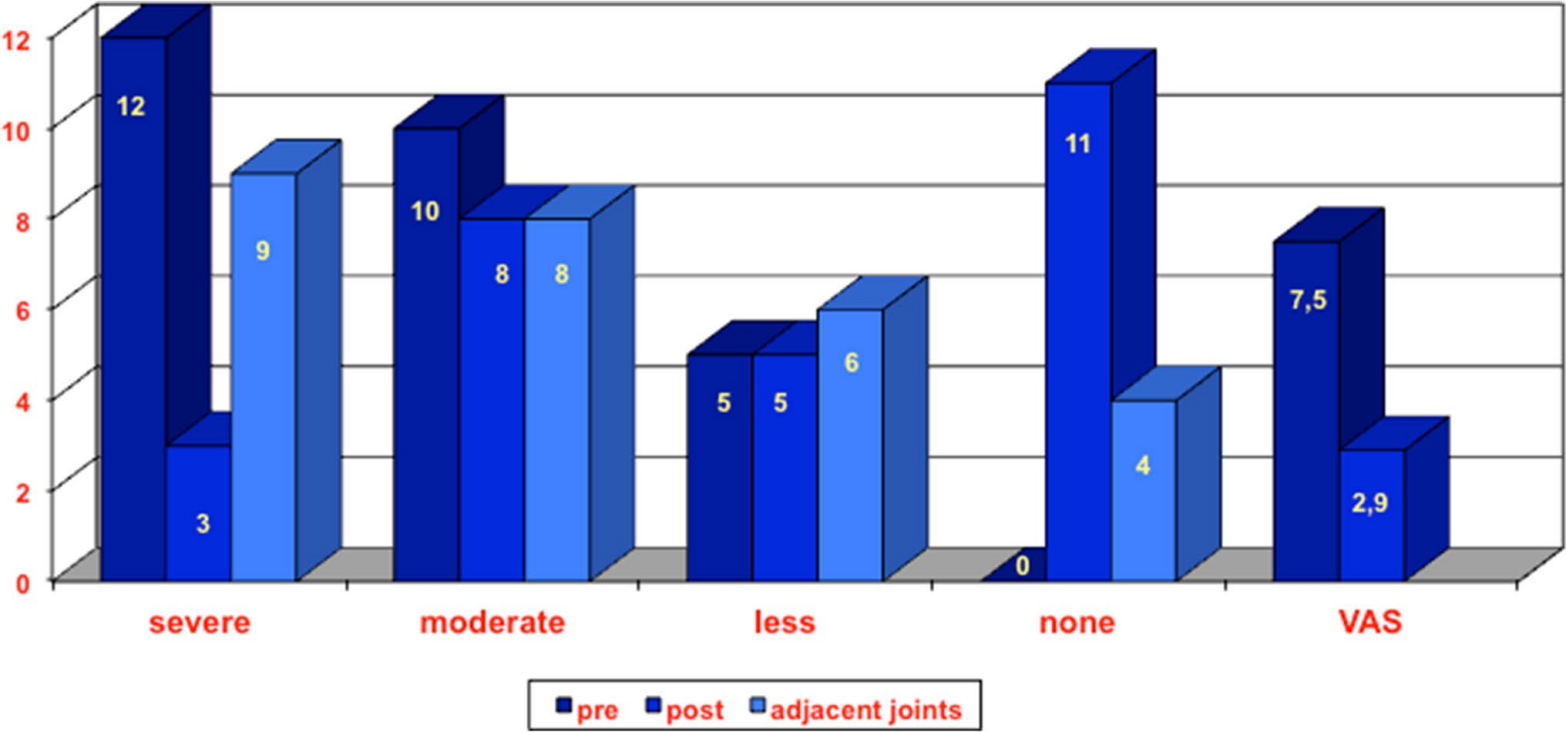
Patient-reported limits for the wrist pre- and postoperative, adjacent joints of the extremity were evaluated separately (third column). Score results at the visual analog scale

The pain improved from 7.5 points preoperatively on the visual analog scale to 2.9 points postoperatively (*p* < 0.05). The significant decrease in pain is mainly caused by the denervation of the interosseus dorsal nerve. The patients still had limitations and projected the postoperative pain onto the hand as a whole. The authors also asked for impairment of the entire arm. The values show these results (Fig. 3).

The patients did not need revision surgery due to renewed ruptures, adhesions, or infections.

## Discussion

Statements on the incidence of rheumatoid extensor rupture in the literature are rare. Depending on the local situation of the thin periarticular structures, rheumatoid arthritis spreads to the tendons at an early stage and leads to detectable changes [6, 13]. Patients tolerate tendon pathologies apparently for a long time if they occur slowly and with peritendinous adhesions [4, 20]. Ruptured tendon sometimes has an artificial function left caused by intact peritendineum. Chung et al. [4] and Barbati et al. [1] describe spontaneous ruptures of damaged extensor tendons as a result of the inflammatory or reactive involvement of the tendons in the disease on the wrist. This matches the experience of the authors.

The primary reconstruction is usually not possible in the ruptured tendon. Publications, therefore, recommend mainly the transfer or coupling of the tendons with an intact extensor tendon [3, 12]. As a second line of defense, many authors favor free transplantation [14, 22]. All tendon operations included in our study were secondary reconstructions with transfer or coupling. A primary suture was never possible, a free graft was not required.

The statistical evaluation in the individual scores revealed poor results. A differentiated evaluation of tendon function was not possible with the scores used. The scores used primarily consider the overall situation of the extremity. Thus, the overall consideration compensated deficits of special care and question. These scores are qualified for patient-related outcome estimation, objective measurements for the affected joint are not possible. There are no finger specific or other scores in the evaluated publications as well.

For this reason, some authors combine different nonspecific scores to make statements on tendon function. Rydholm et al. [17] report in their current publication on testing grip strength in rheumatoid patients. They compared patients' grip strength under basic therapy with developing the disease activity score (DAS28) of pain on the visual analog scale (VAS) and the Health Assessment Questionnaire (HAQ) [21]. Patients with an inflammatory affection of the wrist or tendons to affect the grip forces were excluded in this study.

In rheumatoid patients, tendon ruptures do not occur in isolation. Depending on the duration of the disease, joints, and tendons of the affected extremity are usually already involved. This leads to poor results. Here the authors have to set the bad initial value in relation.

The patients assessed the overall situation of their hand as sufficient (2.7) and at the same time reported a significant improvement in pain (VAS −4.6 points), when the function of the finger extensors was given. Improvement in the Clayton score (+ 10.5 points) was observed, with wrist-dependent improvement in the everyday workload. Patient satisfaction depends on finger and extensor tendon function. In most cases, the postoperative stress on the tendons is reduced with less pain.

Rheumatoid patients functionally tolerate and seemingly compensate partial and complete extensor tendon ruptures for a long time. Therefore, contact with the orthopedic surgeon and operative treatment often takes place late. Rydholm et al. [17] report similar results. There is also a demonstrable difference between observed force and subjective patient assessment of the hand function in its publication. In our cohort, the wrists were involved in averaged 6.4 years, before surgical treatment. Other authors report similar results. When assessing the success of surgery, the global function of the wrist is in the foreground. Subjective patient acceptance is consistently good in assessing the tendon. In our cohort, the EPL tendon was most often affected by 21/62 tendon ruptures (34%). In contrast to this Williamson et al. [21] report, the EDM tendon will rupture first. In our study, only 14/62 tendon ruptures belonged to the EDM tendon (23%).

Previous studies about the postoperative result showed a strong correlation between good tendon function and patient satisfaction in upper limb surgery [2].

Patients do not see the function of the sewn extensor tendon only on its own but evaluate the suitability for daily use of the entire extremity.

**Fig. 4:**
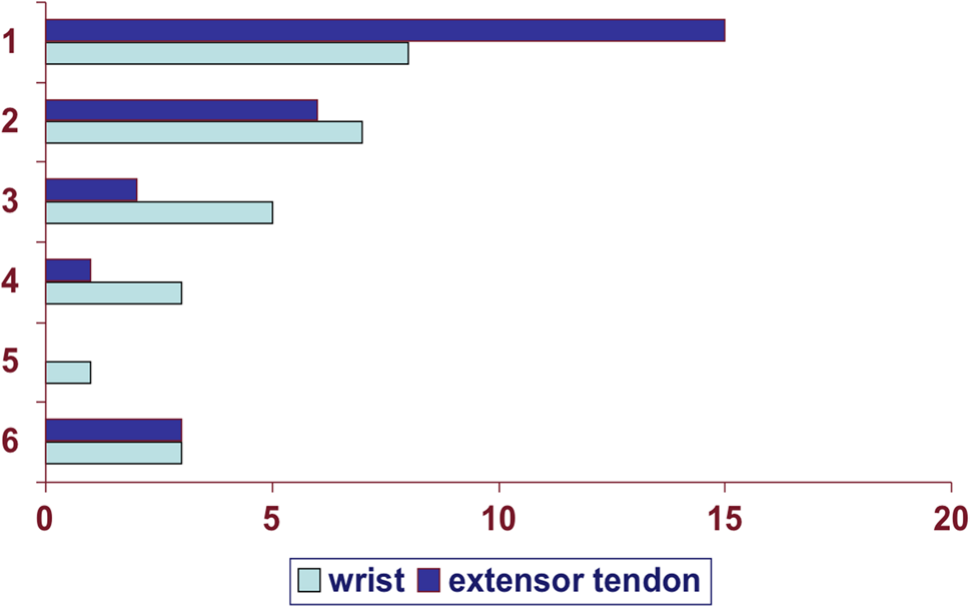
Subjective patient satisfaction according to school notes (1 = best, 6 = worse)

Nevertheless, there are differences between the involved tendons in daily life. Patients with a reconstruction of the EPL tendon still have limitations when using the hand (Fig. 4).

## Conclusion

Rheumatoid patients tolerate and compensate for long term extensor tendon ruptures leading to late clinical presentation. The incidence of an extensor tendon rupture at the wrist is almost 4% and the prevalence of an additional rupture of another tendon is over 50%. Advanced tendon rupture allows only the coupling or transfer of non-affected tendons. The functional scores evaluate the overall situation. A specific score for finger function does not exist. However, the global wrist function is crucial for the daily life of patients, which must be taken into account when planning and assessing therapeutic options.

## Electronic supplementary material

Below is the link to the electronic supplementary material.

Supplementary file1 (DOCX 40 kb)
