# Supplementary material for: Extensor tendon ruptures in rheumatoid wrists
Source: Eur J Orthop Surg Traumatol. 2020 Jul 3;30(8):1499–504. doi: 10.1007/s00590-020-02731-1 (PMC7680316; doi:10.1007/s00590-020-02731-1)
Supplement: Supplementary file 1 — Supplementary file1 (DOCX 40 kb) [file 590_2020_2731_MOESM1_ESM.docx]

**Extensor tendon ruptures in rheumatoid wrists**

**Scores used in the study**

**The QuickDASH – score:**

The QuickDASH Score includes 11 questions, scored 1-5 and is used to assess functional disorders of the upper extremities, especially of the hand, arm, and shoulder. It is not a joint-specific questionnaire but records the function of both extremities as a whole (1).

The QuickDASH score can only be calculated if no more than 1 answer is missing. The calculation is performed according to the following formula:

QuickDASH Score for Disability/Symptoms = (([sum of n answer points]-1) / n) ⋅ 25

The possible score ranges from 0 to 100 points. 0 points represent a complete, unrestricted function of the upper extremities, while 100 points represent the largest possible functional restriction.

<http://www.dash.iwh.on.ca/scoring>

<http://www.dash.iwh.on.ca/sites/dash/public/translations/QuickDASH_English_Australia.pdf>

**The FFbH – score:**

Hannover Functional Questionnaire Backache (FFbH-R) (2).

Questionnaire for the diagnosis of functional disability caused by backache.

The FFbH is an instrument for recording the functional capacity of patients with rheumatoid diseases. The original version was developed especially for rheumatoid arthritis (chronic polyarthritis). However, the FFbH is increasingly used for other chronic joint diseases, where many joints are affected (diseases associated with polyarthritis or chronic polyarticular arthritis).

The FFbH is comparable to the HAQ ("Health Assessment Questionnaire"), which was developed in the 1970s by Jim Fries at Stanford and first published in 1980.

<https://s91a3f7d69a376354.jimcontent.com/download/version/1546535099/module/11311688391/name/FFbH.pdf>

Calculation of FFbH:

Functional capacity (%) = (messured points x 100) : (2 x Number of valid answers)

**The Clayton – score:**

The Clayton-score is a 100-point score (mobility, stability, ligament tension, and pain). Each category awards a multistep evaluation. This score has undergone various further developments, the last update is published in Suk, M et al.: AO Manual. Musculoskeletal Outcomes Measures and Instruments. 2005. ISBN-13: 9783131410610 (3)

90-100 Points excellent

70-89 Points good

60-69 Points acceptable

> 59 Points poor

**The ADL – score:**

The Activities of daily living (ADL) comprise the basic actions that involve caring for one’s self and body, including personal care, mobility, and eating.

ADL-pdf: <https://aging.ufl.edu/files/2012/05/ADLTable.pdf> (4, 5)

Comparison QuickDASH vs. ADL/FFbH - Questionnaire

|  | **QuickDASH** | **ADL/FFbH** |
| --- | --- | --- |
| Question |  |  |
| 1 | Open a tight or new jar | Open a tight or new jar |
| 2 | Do heavy household chores | Do heavy household chores |
| 3 | Carry a shopping bag or briefcase. | Carry a shopping bag |
| 4 | Wash your back | Hygiene |
| 5 | Use a knife to cut food | use cutlery |
| 6 | Recreational activities in which you take some force or impact through your arm, shoulder or hand | Prop oneself up |
| 7 | normal social activity |  |
| 8 | limited in your work or other regular daily activities |  |
| 9 | pain |  |
| 10 | Nerve-palsy: Tingling (pins and needles) in your arm, shoulder or hand. |  |
| 11 | sleeping problems |  |
|  |  | [to](https://dict.tu-chemnitz.de/english-german/to.html) [comb](https://dict.tu-chemnitz.de/english-german/comb.html) [one's](https://dict.tu-chemnitz.de/english-german/one%27s.html) [hair](https://dict.tu-chemnitz.de/english-german/hair.html) |
|  |  | to crop with a scissor |
|  |  | lift up a coin |
|  |  | writing a postcard |

Literature

1. Gummesson C, Ward MM, Atroshi I. The shortened disabilities of the arm, shoulder and hand questionnaire (QuickDASH): validity and reliability based on responses within the full-length DASH. BMC musculoskeletal disorders 7: 44, 2006

2. Raspe H, Hagedorn U, Kohlmann T, Matussek S. Questionnaire for the diagnosis of functional disability caused by backache. German: Funktionsfragebogen FFbH. Ein Instrument zur Funktionsdiagnostik bei polyartikulären Erkrankungen. In: Sigrist J (Hrsg) Wohnortnahe Betreuung Rheumakranker. In: Qualitätssicherung in der Rheumatologie, edited by Rheumatologie DG, Steinkopff: Stuttgart, New York. 1990 / 2008.

3. Clayton ML. Surgical Treatment at the wrist in Rheumatoid Arthritis: A Review of Thirty-Seven Patients. The Journal of bone and joint surgery American volume 47: 741, 1965

4. Mlinac ME, Feng MC. Assessment of Activities of Daily Living, Self-Care, and Independence. Arch Clin Neuropsychol 31: 506, 2016

5. Lawton MP, Brody EM. Assessment of older people: self-maintaining and instrumental activities of daily living. Gerontologist 9: 179, 1969
